# Prevalence and incidence of insufficient physical activity in Brazilian adolescents during the pandemic: data from ConVid Adolescents

**DOI:** 10.1590/1980-549720230049.2

**Published:** 2023-10-27

**Authors:** Nathália Mota Mattos Santi, Crizian Saar Gomes, Danilo Rodrigues Pereira da Silva, Célia Landmann Szwarcwald, Marilisa Berti de Azevedo Barros, Deborah Carvalho Malta

**Affiliations:** I Universidade Federal de Minas Gerais, Faculdade de Medicina, Programa de Pós-Graduação em Saúde Pública - Belo Horizonte (MG), Brasil.; II Universidade Federal de Sergipe, Departamento de Educação Física - São Cristóvão (SE), Brasil.; III Universidad Autónoma de Chile, Faculty of Health Sciences - Providencia, Chile.; IV Fundação Oswaldo Cruz, Instituto de Comunicação e Informação Científica e Tecnológica em Saúde - Rio de Janeiro (RJ), Brasil.; V Universidade Estadual de Campinas, Faculdade de Ciências Médicas, Departamento de Saúde Coletiva - Campinas (SP), Brasil.; VI Universidade Federal de Minas Gerais, Escola de Enfermagem, Departamento de Enfermagem Materno Infantil e Saúde Pública - Belo Horizonte (MG), Brasil.

**Keywords:** COVID-19, Pandemic, Adolescent, Physical activity, Physical inactivity, COVID-19, Pandemia, Adolescente, Atividade física, Inatividade física

## Abstract

**Objective:**

To evaluate the prevalence and incidence of insufficient physical activity in Brazilian adolescents and identify the most affected subgroups during the pandemic.

**Methods:**

This study used data from the “ConVid Adolescents - Behavior Survey”, which evaluated the behavior of 9,470 Brazilian adolescents during the period of social restriction due to the COVID-19 pandemic in 2020, through a self-administered online questionnaire. Participants were invited through a virtual “snowball” sampling procedure. Information was reported on the frequency of physical activity before and during the pandemic. The exposure variables used were gender, age group, race/skin color, region of Brazil, type of school, maternal education, financial difficulties during the pandemic, and social restrictions. Logistic regression models were used.

**Results:**

Adolescents engaged in less physical activity during the pandemic, as the prevalence of insufficient physical activity increased from 71.3% in the previous period to 84.3% during the pandemic. The incidence of insufficient physical activity during the pandemic was 69.6%. The subgroups of adolescents most affected were those who self-declared as black or with dark skin color, reported financial difficulties during the pandemic, lived in the Southeast and South regions of the country, and practiced intense or complete social distancing.

**Conclusion:**

High incidences of insufficient physical activity were observed among Brazilian adolescents during the COVID-19 pandemic. It is recommended that further studies explore periods after those analyzed to identify the behavioral dynamics of adolescents upon returning to in-person activities.

## RESUMO

**Objetivo:**

Avaliar a prevalência e a incidência de atividade física insuficiente em adolescentes brasileiros e identificar os subgrupos mais afetados durante a pandemia.

**Métodos:**

Este estudo utilizou dados da *ConVid Adolescentes - Pesquisa de Comportamentos*, que avaliou, por meio de um questionário online autopreenchido, o comportamento de 9.470 adolescentes brasileiros no período de restrição social, em virtude da pandemia da COVID-19 em 2020. Os participantes foram convidados por meio de um procedimento de amostragem em cadeia denominado “bola de neve” virtual. Foram relatadas informações sobre a frequência de atividade física antes e durante a pandemia. As variáveis de exposição utilizadas foram sexo, faixa etária, raça/cor da pele, região do Brasil, tipo de escola, escolaridade materna, dificuldades financeiras durante a pandemia e restrições sociais. Foram utilizados modelos de regressão logística.

**Resultados:**

Os adolescentes praticaram menos atividade física durante a pandemia, visto que a prevalência de atividade física insuficiente aumentou de 71,3% no período anterior para 84,3% durante a pandemia. A incidência de atividade física insuficiente durante a pandemia foi de 69,6%. Os subgrupos de adolescentes mais afetados foram os que se autodeclararam da raça/cor da pele preta, que relataram dificuldades financeiras durante a pandemia, residentes nas regiões Sudeste e Sul do país e que realizaram restrição social intensa e total.

**Conclusão:**

Altas incidências de atividade física insuficiente foram observadas entre adolescentes brasileiros durante a pandemia da COVID-19. Recomenda-se que novos estudos explorem períodos posteriores aos analisados para identificar a dinâmica comportamental dos adolescentes a partir do retorno das atividades presenciais.

## INTRODUCTION

COVID-19 has brought several developments to health, the economy, politics, and society since it was declared a pandemic, on March 11^th^, 2020, by the World Health Organization (WHO)^1^. In Brazil, a complex epidemiological panorama was faced due to the need for public health measures to mitigate the spread of the virus and the austerity policies that were already being implemented before the outbreak of the pandemic in the country^2^ ^,^ ^3^.

During the first wave of COVID-19, different social distancing measures were imposed by government and health authorities, which include closing spaces, suspending some types of commerce, banning and canceling social events^4^. With social distancing, families were encouraged to stay in their homes; however, such measures have produced fewer opportunities for an active lifestyle^5^.

Some groups, such as teenagers and young people, were particularly affected by the effects of social distancing^6^. In Brazilian adolescents, there was an increase in sedentary behavior and consumption of unhealthy foods, worsening sleep quality, self-reported sadness, irritability, and loneliness due to the social distancing measures implemented^6^ ^,^ ^7^. 

Studies conducted in Saudi Arabia, Canada, and Ireland indicate a reduction in the practice of physical activity (PA) in this population group during the pandemic^8^ ^,^ ^9^ ^,^ ^10^. In fact, even before the advent of the pandemic, low rates of PA in adolescents were already considered a serious public health problem^11^. Global estimates indicate that, before the pandemic, 81% of adolescents were insufficiently active^12^. Brazil follows the same trend, with a low proportion of adolescents who meet PA guidelines, around 28% only^13^. However, the literature is very consistent regarding the health benefits provided by the practice of PA^14^ ^,^ ^15^ ^,^ ^16^ ^,^ ^17^, and recent WHO guidelines and the Physical Activity Guide for the Brazilian Population recommend that adolescents accumulate an average of 60 minutes of practice per day, as well as to include muscle and bone strengthening activities at least 3 days a week^17^ ^,^ ^18^. 

Studies indicate that the practice of PA in adolescence is related to a greater probability of practicing PA in adulthood^19^ ^,^ ^20^ ^,^ ^21^. As reported by McGuire et al. (2001), although several weeks or a few months of physical inactivity probably do not cause a sudden onset of metabolic disease, the abrupt interruption of PA can trigger changes in insulin sensitivity and muscle loss^22^. Additionally, a sudden decrease in PA can negatively impact depressive symptoms, anxiety, fatigue, and energy levels^23^.

In this context, as adolescence is a period of acquiring new lifestyle habits that will be determinants of current and future health and that may constitute risk factors for diseases^24^ ^,^ ^25^ ^,^ ^26^, this likely change in PA practice during the pandemic should be investigated and understood, given the difficulty in staying active due to social distancing.

Most of the studies already published in the country and around the world that evaluate changes in individuals’ lifestyles during the pandemic target the adult population, with few studies conducted with adolescents. Furthermore, to date, no national studies have been found that identify which subgroups in this age range were most affected by social distancing. The identification of these subgroups could collaborate in the development of effective measures to minimize the damage caused by the pandemic.

In this sense, the objectives of the present study were to evaluate the prevalence and incidence of insufficient PA in Brazilian adolescents and identify the subgroups most affected during the pandemic, based on the analysis of data from *ConVid Adolescentes* - Behavior Survey.

## METHODS

### Sampling

This is a cross-sectional epidemiological study that used data from *ConVid Adolescentes* - Behavior Survey^27^. *ConVid Adolescentes* is a survey that aimed to evaluate the behavior of Brazilian adolescents during the COVID-19 pandemic, carried out across the country by the Oswaldo Cruz Foundation (Fundação Oswaldo Cruz - Fiocruz) in partnership with Universidade Federal de Minas Gerais (UFMG) and Universidade Estadual de Campinas (Unicamp).

The questionnaire was prepared using the RedCap application (Research Electronic Data Capture) and self-completed by teenagers online, after consent from their guardian and the teenagers themselves, on a smartphone or computer with internet access.

Sampling was carried out using the “virtual snowball” method. Project coordinators selected researchers from different Brazilian states to begin the process, sending the research link to parents or guardians of teenagers. In addition to presenting the research, the message with the link accompanied a request for it to be shared with the network of contacts of those who received it. Upon receiving the invitation to participate in the research, parents or guardians were asked the following question: “Do you have children or are you responsible for young people aged 12 to 17yo?” Only those who responded affirmatively received the Informed Consent with explanations about the study, a link for contacts and clarifications about the research and the request for consent to participate from the minor under their responsibility. After acceptance of the Informed Consent by the responsible adult, the adolescent received the Free and Informed Assent. Only after accepting the Assent Form did the respondent begin filling out the questionnaire. Furthermore, the research coordination team contacted public and private schools, state and municipal education departments, via institutional email. The institutions that participated in the research sent the electronic questionnaires to the students’ guardians.

The anonymity of responses was guaranteed, and it was not possible to identify respondents in any way. All procedures were approved by the National Research Ethics Commission (Decision Number: 4.100.515).

Since network sampling is not probabilistic, post-stratification procedures were used to obtain a representative sample of the population of adolescents with the same distribution by region of residence, gender, age range (12-15; 16-17) and type of school (public; private), based on data from the National School Health Survey (*Pesquisa Nacional de Saúde Escolar* - PeNSE, 2015) of the Brazilian Institute of Geography and Statistics (*Instituto Brasileiro de Geografia e Estatística* - IBGE) carried out in partnership with the Ministry of Health^28^.

Data collection took place between June 27^th^ and September 17^th^, 2020. The sample reached 9,470 adolescents aged 12 to 17 years old from all Brazilian states, with questionnaires with missing information for PA (n=89) being excluded from the analyses. For incidence analyses, individuals who had insufficient PA (<300 minutes/week) before the pandemic (n=6,686) were excluded. Therefore, for analyses related to incidence, the final sample consisted of 2,695 adolescents.

### Variables

The questionnaire used is available on the research website (https://convid.fiocruz.br/) and was based on questions validated in health surveys previously applied in the country, such as the National Health Survey, and monitored by the Surveillance of Risk and Protective Factors for Chronic Diseases by Telephone Survey (*Vigilância de Fatores de Risco e Proteção para Doenças Crônicas por Inquérito Telefônico* - Vigitel)^29^ ^,^ ^30^_._

### Insufficient physical activity

PA was characterized by the time spent in any PA daily by adolescents. This variable was assessed by the following questions: “Before the coronavirus pandemic, on how many days did you practice PA for at least 60 minutes (1 hour) per day? *E.g*.: playing sports, playing ball, cycling, walking, running, taking Physical Education classes, going to school walking or cycling (Add up all the time you spent on any type of PA, each day).”; “During the pandemic, on how many days did you practice PA for at least 60 minutes (1 hour) per day? (Add up all the time you spent on any type of PA, each day).”

Insufficient PA was considered when adolescents reported performing less than 5 days of PA lasting at least 60 minutes^13^.

### Exposure variables

The exposure variables analyzed were: gender (female; male); age group (12 to 15; 16 to 17 years old); race/skin color (white; black; brown; others); type of school (private; public); mother’s education level (incomplete high school; complete high school; complete higher education); financial difficulties during the pandemic (yes; no); region (North; Northeast; Southeast; South; Central-West); and social restriction (no restriction; little restriction; intense restriction; total restriction). No restriction was considered when the teenager responded “I didn’t do anything, I led a normal life”; little restriction for the answer “I tried to take care, stay away from people, reduce contact a little, not visit the elderly, but I kept going out”; intense restriction when choosing the option “I stayed at home most days, going to close family members’ houses, shopping at supermarkets and pharmacies”; and total restriction when he responded “I stayed strictly at home, only leaving for health care needs”.

### Data analysis

The sample characteristics were described using relative frequencies and 95% confidence intervals (95%CI). Initially, the prevalence (95% CI) of the outcome before and during the pandemic was calculated for the total sample and according to the exposure variables. Differences between prevalence rates before and during the pandemic were considered significant when there was no overlap in the 95%CI of the prevalence rates in question. Subsequently, to estimate the incidence of the outcome, adolescents who were insufficiently active before the pandemic were excluded. To verify the possible subgroups most affected by the outcome, the odds ratio was used as a measure of association, obtained through logistic regression with robust variance. The subgroups that presented p<0.20 in the univariate analyses were included in the multivariate model. In the final model, those with p<0.05 were considered to be the most affected subgroups. All analyses were performed using Stata 14.1 software and post-stratification weights were considered.

## RESULTS

Table 1 presents the characteristics of the total sample. Of the 9,470 adolescents assessed, 50.3% were girls; 67.7% aged between 12 and 15 years; 46.6% were of brown race/skin color; 85.9% studied in public schools; and 41.2% lived in the Southeast region. Around a third of teenagers responded that they experienced financial difficulties during the pandemic and 45.6% said they had complied with intense social restrictions.

**Table 1.** Sample characteristics, *ConVid Adolescentes*, 2020.

Characteristics	Total (n=9,470)
	% (95%CI)
Gender	
Male	49.7 (48.1-51.4)
Female	50.3 (48.6-51.9)
Age range (years)	
12-15	67.7 (66.3-69.1)
16-17	32.3 (30.9-33.7)
Race/skin color	
White	40.1 (38.5-41.7)
Black	9.7 (8.8-10.7)
Brown	46.6 (44.9-48.3)
Others	3.6 (3.0-4.4)
Type of school	
Public	85.9 (85.1-86.7)
Private	14.1 (13.3-14.9)
Maternal education	
Incomplete high school	32.6 (30.9-34.2)
Complete high school	33.8 (32.1-35.5)
Complete higher education	33.6 (32.1-35.2)
Region	
North	9.1 (8.6-9.7)
Northeast	28.4 (26.4-30.4)
Southeast	41.2 (39.7-42.8)
South	13.6 (13.0-14.2)
Central-West	7.7 (7.1-8.4)
Financial difficulties during the pandemic	
No	66.1 (64.5-67.7)
Yes	33.9 (32.3-35.5)
Social restriction	
No restriction	4.7 (4.0-5.4)
Little restriction	23.8 (22.4-25.3)
Intense restriction	45.6 (43.9-47.3)
Total restriction	25.9 (24.5-27.4)

95%CI: 95% confidence interval.

In the analysis of the prevalence of insufficient PA before and during the pandemic (Table 2), the differences between them were considered significant when there was no overlap in the 95%CI of the prevalences considered. Thus, an increase from 71.3 to 84.3% in the prevalence of insufficient PA was observed, that is, the number of insufficiently active adolescents increased during the isolation period, with this pattern being observed in all subgroups, except among adolescents who declared themselves to be of indigenous or yellow race/skin color (others) and that they did not make any social restrictions.

**Table 2.** Prevalence of insufficient physical activity before and during the COVID-19 pandemic among Brazilian adolescents according to sociodemographic characteristics.

Characteristics	Insufficient physical activity	
	Before (%; 95%CI)	During (%; 95%CI)
Total	71.3 (69.8-72.8)	84,3 (83,0-85,4)
Gender		
Male	67.5 (65.0-69.9)	84.9 (82.9-86.7)
Female	75.1 (73.4-76.7)	83.6 (82.1-85.1)
Age range (years)		
12-15	72.2 (70.3-74.1)	85.1 (83.5-86.6)
16-17	69.4 (67.2-71.5)	82.5 (80.6-84.2)
Race/skin color		
White	71.7 (69.6-73.7)	83.6 (81.8-85.2)
Black	68.2 (62.5-73.3)	87.5 (84.1-90.3)
Brown	71.7 (69.3-73.9)	84.6 (82.6-86.4)
Others (indigenous/yellow)	71.0 (62.6-78.2)	78.7 (68.8-86.1)
Type of school		
Public	71.5 (69.0-73.9)	83.7 (81.5-85.7)
Private	71.3 (69.6-72.9)	84.4 (82.9-85.7)
Maternal education		
Incomplete high school	74.7 (72.0-77.3)	87.2 (85.2-89.0)
Complete high school	71.3 (68.5-74.0)	83.2 (80.7-85.5)
Complete higher education	67.6 (64.9-70.1)	83.8 (81.6-85.7)
Financial difficulties during the pandemic		
No	71.6 (69.7-73.4)	82.4 (80.7-83.9)
Yes	70.6 (68.1-73.1)	87.9 (86.1-89.4)
Region		
North	70.2 (67.7-72.6)	78.1 (75.7-80.4)
Northeast	73.2 (68.9-77.1)	81.8 (78.1-85.0)
Southeast	71.9 (69.9-73.8)	87.6 (86.2-88.9)
South	68.2 (66.4-70.0)	84.8 (83.4-86.1)
Central-West	68.0 (63.7-72.1)	81.8 (78.1-85.1)
Social restriction		
No restriction	84.3 (78.9-88.6)	89.7 (85.5-92.7)
Little restriction	69.6 (66.3-72.7)	83.5 (80.8-86.0)
Intense restriction	70.1 (67.9-72.3)	84.0 (82.1-85.7)
Total restriction	72.5 (69.6-75.3)	84.4 (82.0-86.5)

95%CI: 95% confidence interval.

Table 3 presents the incidence of insufficient PA during the pandemic and the most affected subgroups. The subgroups that presented p<0.20 in the univariate analyses were included in the multivariate model. Approximately 70% of teenagers who were active before the pandemic became insufficiently active during the isolation period. Adolescents who self-declared to be black (ORadj 2.00; 95%CI 1.11-3.60), who reported financial difficulties during the pandemic (ORadj 1.85; 95%CI 1.37-2.48), residents in the Southeast (ORadj 2.78; 95%CI 2.02-3.84) and South (ORadj 2.16; 95%CI 1.57-2.98) regions of the country and who underwent intense (ORadj 3.41; 95%CI 1.48-7.91) and total (ORadj 2.58; 95%CI 1.09-6.10) social restrictions showed a higher incidence of insufficient PA during the pandemic.

**Table 3.** Incidence of insufficient physical activity during the COVID-19 pandemic according to sociodemographic characteristics, *ConVid Adolescentes*, 2020.

Characteristics	% (95%CI)	Univariate model	Multivariate model
		OR (95%CI)	ORadj (95%CI)
Total	69.6 (66.7-72.4)		
Gender			
Male	71.0 (66.6-74.9)	1	---
Female	67.8 (64.0-71.4)	0.86 (0.66-1.12)	---
Age range (years)			
12-15	69.3 (65.4-73.0)	1	---
16-17	70.1 (66.0-73.9)	1.04 (0.80-1.35)	---
Race/skin color			
White	69.2 (64.9-73.1)	1	1
Black	82.2 (74.6-87.9)	2.06 (1.26-3.36)	2.00 (1.11-3.60)
Brown	67.9 (63.1-72.3)	0.94 (0.71-1.25)	1.11 (0.79-1.57)
Others	57.7 (42.3-71.7)	0.61 (0.32-1.16)	0.53 (0.26-1.06)
Type of school			
Public	71.8 (66.8-76.3)	1	---
Private	69.2 (65.9-72.4)	0.88 (0.67-1.17)	---
Maternal education			
Incomplete high school	72.7 (67.0-77.8)	1	---
Complete high school	65.4 (59.7-70.6)	0.71 (0.49-1.02)	---
Complete higher education	74.8 (70.4-78.6)	1.11 (0.78-1.58)	---
Financial difficulties during the pandemic			
No	64.6 (60.8-68.3)	1	1
Yes	78.9 (75.0-82.4)	2.05 (1.55-2.70)	1.85 (1.37-2.48)
Region			
North	53.7 (48.8-58.5)	1	1
Northeast	61.5 (52.6-69.7)	1.38 (0.91-2.08)	1.36 (0.86-2.14)
Southeast	79.3 (75.9-82.3)	1.48 (2.50-4.34)	2.78 (2.02-3.84)
South	72.3 (69.0-75.3)	2.25 (1.75-2.88)	2.16 (1.57-2.98)
Central-West	62.1 (53.8-69.6)	1.41 (0.95-2.08)	1.13 (0.75-1.73)
Social restriction			
No restriction	45.1 (29.8-61.3)	1	1
Little restriction	65.5 (59.4-71.1)	2.31 (1.14-4.70)	2.35 (1.00-5.49)
Intense restriction	73.4 (69.0-77.4)	3.37 (1.68-6.74)	3.41 (1.48-7.91)
Total restriction	68.9 (63.1-74.2)	2.70 (1.33-5.50)	2.58 (1.09-6.10)

95%CI: 95% confidence interval; OR: odds ratio.

Note. 1. Only adolescents with sufficient physical activity before the pandemic were included in the incidence analysis (n=2,695). 2. The subgroups that presented p<0.20 in the univariate analyses were included in the multivariate model (race/color, financial difficulties during the pandemic, region, social restrictions).

## DISCUSSION

The *ConVid Adolescentes* study analyzed data among Brazilian adolescents during the pandemic and found that approximately seven in ten adolescents who were active before the pandemic were no longer sufficiently active during the pandemic period. In all subgroups, the majority of active adolescents became insufficiently active. The highest incidences of insufficient PA were observed among adolescents who declared themselves black, who reported financial difficulties during the pandemic, residing in the Southeast and South regions of the country and who underwent intense and total social restriction.

This analysis represents the period in which the first wave of COVID-19 occurred in Brazil, with a rapid spread of the disease throughout the country and the adoption of strict social distancing measures, such as closing schools, workplaces, and some types of business^2^. 

The findings of the present study indicate that the prevalence of insufficient PA increased by more than 10 percentage points during the COVID-19 pandemic among Brazilian adolescents, except among those who declared themselves to be of indigenous or yellow race/skin color (others) and who made no social restrictions. During the pandemic, the recommendation for indigenous peoples was to remain in their villages as a protective measure, which may explain the results found in this research, as indigenous communities already experience social distancing in relation to other communities for traditional and cultural reasons, and these measures were reinforced by the government^31^.

The results revealed may have repercussions on the quality of life of these subgroups, since abrupt interruption of PA, even for a short period, is associated with negative health outcomes^23^. The increase in insufficient PA among adolescents during the pandemic is worrying, as current PA habits can predict future trends in whether or not this practice is adhered to^19^ ^,^ ^21^ ^,^ ^32^_._

This result corroborates other research carried out with adolescents in different countries. A study evaluated the PA practice of a total of 726 adolescents from Italy, Spain, Brazil, Chile, and Colombia, and found that the prevalence of adolescents considered physically inactive (<300 minutes of PA/week) was 73% before social isolation and 79.5% in this period^33^. In Ireland^10^ and Saudi Arabia^8^, 49.7 and 59% of adolescents, respectively, reported performing less PA during the lockdown period, that is, they reduced the number of days on which they practiced at least 60 minutes of moderate to vigorous PA daily. Among Italian teenagers, the time dedicated to sports during social distancing measures decreased significantly by 2.3 hours per week^34^. Such findings can be explained by the closure of places such as schools, gyms, parks, squares, clubs and other spaces with crowds of people, in addition to the limitation of socializing with friends during the period of social isolation^35^. It is noteworthy that the school is established as a strategic space for creating leisure, sports and PA opportunities for adolescents^36^ ^,^ ^37^, and the closure of these institutions made it difficult for students to continue practicing PA.

Taking into account that most adolescents generally practice PA outside the home, such as walking to school, physical education classes, recess, extracurricular and group activities^38^ ^,^ ^39^, this situation may justify the higher incidences of insufficient PA found in this research among adolescents who experienced restrictive measures intensely or completely during the pandemic. Our data corroborates those of a literature review that found a reduction in the practice of PA in children and adolescents from different countries due to the social restrictions imposed by the pandemic in 16 of the 17 studies analyzed^40^. Caldwell et al. found regional differences in Canada: children and young people who lived in regions with fewer public health restrictions practiced more PA than those who experienced more restrictive policies^41^.

In this research, adolescents from the South and Southeast regions of Brazil had a higher incidence of physical inactivity compared to the North region, which can be explained by the fact that populations from the South and Southeast had greater adherence to social restriction measures, as pointed out by some studies^42^ ^,^ ^43^. A survey analyzed remote work in the context of the pandemic and found that the regions where the most workers adopted this modality were the Southeast and South regions of the country^43^. With parents working from home, monitoring their children is easier and the need to leave the house can be reduced. However, a possible inadequacy of the sample in the adjustments by region in this research may have influenced these results.

When considering race and financial difficulty as proxies for socioeconomic status, the present study confirms the influence of socioeconomic level on the practice of PA^44^ ^,^ ^45^. Adolescents who declared themselves to be black and who had financial difficulties during the pandemic had a higher incidence of insufficient PA. This association can be explained by inequalities in access and fewer opportunities to be physically active in different spaces among those with lower purchasing power, which further exacerbates health inequities^17^ ^,^ ^20^. It is possible that adolescents who lived in houses with outdoor space or other large spaces, such as backyards and garages, decreased their PA levels less than those who did not have these possibilities, such as those who lived in communities and outskirts, where there is high population density, with the majority being black^46^ ^,^ ^47^. In Spain, there was also a tendency toward a decrease in PA engagement as the socioeconomic level decreased^37^. The findings of this study reinforce what was also pointed out in other studies, that COVID-19 highlighted the vulnerabilities and inequalities that exist in society, exacerbating pre-existing racial and socioeconomic inequalities, especially between black and brown people^48^ ^,^ ^49^. Such disparities are fueled by social determinants of health, in a society with long-standing deep structural, racial and socioeconomic inequalities^49^.

Regarding the limitations of this study, some aspects should be mentioned. Due to the research design itself, some population groups may be underrepresented, such as low-income people, those with difficulty accessing the internet and those with a lower level of education. However, sample calibration using PeNSE data to generate adequate estimates reduced this limitation. Furthermore, the restricted period of data collection must be considered when interpreting the findings. PA domains were combined into a single question; Furthermore, self-reported behaviors are susceptible to memory bias and classification errors in categorical responses, such as those related to frequency and duration of PA. Finally, the number of insufficiently active adolescents may have been overestimated by the categorization of the variable that considered as active only those who practiced 60 minutes or more of PA on 5 days of the week.

It is noteworthy that this is, to date, the first nationally representative study to quantify the incidence of insufficient PA among adolescents and to identify the subgroups most affected during the COVID-19 pandemic, which could guide managers toward actions and policies to effective public health measures for this and possible future pandemic scenarios.

In summary, a high incidence of insufficient PA, with an impact on the increased prevalence of this behavior, was observed among Brazilian adolescents during the COVID-19 pandemic. The most affected population subgroups were those who declared themselves to be black, who reported financial difficulties during the pandemic, who lived in the Southeast and South regions of the country and who underwent intense and total social restrictions, which should be prioritized in intervention strategies.

Simply ending social distancing measures and resuming daily activities may not be enough to reverse the scenario identified in this study, as the worsening of the outcome observed is not an exclusive product of the pandemic context we are experiencing. The findings of this study reflect the social context of the country, marked by structural inequities that require profound changes in socioeconomic patterns^2^ ^,^ ^4^. The pandemic, understood as a syndemic because it interacts with and aggravates pre-existing complications due to its intertwining with social and environmental determinants, has brought to light discussions that should be a priority on political agendas^50^.

The search for improving the quality of life among adolescents must be present in the main health promotion strategies, with intra- and intersectoral actions being urgent, as well as the intensification of public social protection policies. These actions must involve strategies to encourage PA at home, outdoors and in school environments. In possible future pandemic scenarios, PA strategies in indoor and outdoor environments should be encouraged and organized to avoid crowds, as well as a return to PA habits prior to the pandemic period. It is recommended that new studies be conducted to evaluate adolescents’ behaviors in the years following the pandemic.
